# Yoga as a Preventive Intervention for Cardiovascular Diseases and Associated Comorbidities: Open-Label Single Arm Study

**DOI:** 10.3389/fpubh.2022.843134

**Published:** 2022-06-13

**Authors:** Kaushal Sharma, Indranill Basu-Ray, Natasha Sayal, Ariana Vora, Sridhar Bammidi, Rahul Tyagi, Shweta Modgil, Parul Bali, Paramvir Kaur, Atul Kumar Goyal, Deepak Kumar Pal, Harshita Arvind, Khushboo Jindal, Vincy Garg, Bandu Matyal, Neha Thakur, Amit Chhikara, Navneet Kaur, Preety Maanju, Kulsajan Singh Bhatia, Viraaj Pannu, Vanita Gupta, Neeru Malik, Rakesh Malik, Raman Kumar, Ravneet Kaur, Vinod Bhatt, Ashish Bhalla, Manju Mohanty, Gurmeet Singh, Suresh Kumar Sharma, Madhava Sai Sivapuram, Deepali Mathur, Dibbendu Khanra, Akshay Anand

**Affiliations:** ^1^Neuroscience Research Lab, Department of Neurology, Post-graduate Institute of Medical Education and Research, Chandigarh, India; ^2^Centre for Systems Biology and Bioinformatics, Panjab University, Chandigarh, India; ^3^Department of Cardiology, St. Francis Hospital, Memphis, TN, United States; ^4^Department of Cardiology, All India Institute of Medical Sciences, Virbhadra Marg, Rishikesh, India; ^5^Department of Cardiology, Swami Vivekananda Yoga Anusandhana Samsthana, Union Territory (SVYASA), Bengaluru, India; ^6^Department of Physical Medicine and Rehabilitation, Massachusetts General Hospital, Boston, MA, United States; ^7^Department of Zoology, Panjab University, Chandigarh, India; ^8^Department of Biophysics, Panjab University, Chandigarh, India; ^9^Department of Otolaryngology, Post-graduate Institute of Medical Education and Research, Chandigarh, India; ^10^Department of Bioinformatics, Jaypee University, Shimla, India; ^11^Department of Physical Education, Panjab University, Chandigarh, India; ^12^Government Medical College, Chandigarh, India; ^13^Department of Health, UT, Chandigarh, India; ^14^Department of Physical Education, Dev Samaj College of Education, Chandigarh, India; ^15^Department of Physical Education and Sports, Panjab University, Chandigarh, India; ^16^Yoga Federation of India, Panchkula, India; ^17^Department of Internal Medicine, Post-graduate Institute of Medical Education and Research, Chandigarh, India; ^18^Department of Neurosurgery, Post-graduate Institute of Medical Education and Research, Chandigarh, India; ^19^Department of Statistics, Panjab University, Chandigarh, India; ^20^Dr. Pinnamaneni Siddhartha Institute of Medical Sciences and Research Foundation, Vijayawada, India; ^21^School of Biotechnology, KIIT University, Bhubaneswar, India

**Keywords:** cardiovascular diseases, yoga, body mass index, cholesterol levels, triglycerides

## Abstract

**Aim:**

Common Yoga Protocol (CYP) is a standardized yoga protocol authored by experts from all over the world under the aegis of the Ministry of AYUSH, Ayurveda, Yoga and Naturopathy, Unani, Siddha, Sowa Rigpa and Homeopathy (AYUSH). The potential of CYP can be determined as a cost-effective lifestyle modification to prevent the risk of developing cardiovascular diseases (CVD).

**Methods:**

In this prospective trial, we compared the effect of CYP at baseline and after 1 month. A total of 374 yoga-naïve participants performed CYP under the supervision of experienced trainers. Physiological [body mass index (BMI), blood pressure, percent oxygen saturation], biochemical (fasting blood glucose and lipid profile), and neurocognitive parameters were measured before and after the intervention.

**Results:**

At day 30 of yoga practice, serum levels of low-density lipoprotein (LDL), total cholesterol (TC), and high-density lipoprotein (HDL) were found significantly improved as compared to the baseline levels observed at the time of enrollment. Similarly, the lipid profile was also obtained from experienced trainers and found to be significantly different from those of yoga-naïve volunteers. When the intervention was compared between the healthy yoga-naïve participants with yoga-naïve participants suffering from medical issues, it was found that cholesterol profile improved significantly in the healthy-naive group as compared to the diseased group (hypertension, diabetes, underwent surgery, and CVD).

**Conclusion:**

These results highlight the need for further research to better understand the effects of yoga on the primary prevention of CVD.

## Introduction

Cardiovascular disease (CVD) is the leading cause of mortality in India as well as across the globe (1, 2). Accumulating evidence indicates that CVD affects the Indian population at a much early age and a decade earlier than the European population (3, 4). It has been estimated that around 52% of Indians below 70 years of age die due to CVDs. However, CVDs affect only 23% of people below this age who hail from western countries (5). The World Health Organization (WHO) global status report on non-communicable diseases reported 17.3 million cardiovascular deaths in 2008, of which 7.3 million were due to myocardial infarction and 6.2 million were due to stroke (2). Furthermore, it has been seen that CVDs are more prevalent in low-income countries like India as compared to middle- and high-income countries (6, 7). Though the reason is not fully understood, it may be due to distinct biological processes, social factors, and their interactions that may be responsible for an increased death rate in India. Given the light shed on the proportion of fatality cases reported in India caused due to CVDs, it appears that it is going to pose a major socio-economic challenge to healthcare industries as well as caregivers (8). Hence, it is important to understand the biological and social determinants and identify therapeutic strategies that are cost-effective and yield promising results. In addition, hyperlipidemia is associated with atherosclerosis and diabetes, and it is known to increase with a sedentary lifestyle, stress, age, and an unhealthy diet (9, 10). According to the consensus statement of the Lipid Association of India (2016), there has been an exponential increase in early-onset atherosclerotic cardiovascular disease (ASCVD) (11). CVD has established risk factors including hyperlipidemia, poor dietary habits, physical inactivity, and smoking (12, 13).

A systematic review conducted in 2017 revealed that 50% of the people suffering from type-2 diabetes mellitus (T2DM) died due to CVD (14). Among the specific diseases, coronary artery diseases (CAD) were the most prevalent CVDs that occurred in T2DM patients whereas stroke was the least prevalent. The preponderance of males affected with CVD was more as compared to females. Other than T2DM, age and obesity are deemed important risk factors associated with CVD. The findings of this review reveal that CVD was the principal cause of death among patients with T2DM with CAD having the highest prevalence. Similarly, another investigation by Straka et al. (15) demonstrated that T2DM patients who came for follow-up for 1 year had the highest risk of heart failure, followed by stroke, myocardial infarction, and the lowest risk of CAD. It can therefore be presumed that T2DM is a major risk factor for CVD. Figures indicate that 8% of people who died with T2DM were in the age group of 20–79 years which represents 5 million deaths globally (16). There are also reports indicating that nearly 50% of the people aged 20–79 years are unaware of their disease hence proper diagnosis and timely treatment remains a major factor in increased rate of mortality (17). Particularly, people from the African region constituting 66.7% of its population, lack awareness about the disease and its associated risks hence the disease remains undiagnosed (18). It is imperative to understand the importance of other effective interventions that are likely to reduce CVD burden otherwise it may lead to a substantial loss in the national economy.

Yoga has already been proven an effective and affordable means of intervention in health care and it may reduce the burden of CVD as well. Numerous studies have reported the effects of yoga on biochemical parameters such as glucose (19), uric acid (20), and total protein (21). In a study conducted on individuals with type 2 diabetes and dyslipidemia, yoga practice was found to be superior to sulphonylurea treatment in improving total cholesterol (TC), triglycerides (TG), and low-density lipoprotein (LDL) levels (22). However, the yoga practice was not standardized. A 2016 meta-analysis of yoga for type 2 diabetes showed improved fasting glucose, lipid profile, and blood pressure with yoga practice as compared to standard care (23).

This is the first study conducted to assess the effectiveness of the AYUSH yoga protocol which does not belong to any particular yoga school. This is a consensus protocol evolved after the Delphi round of discussions comprising all major schools of Yoga in India, inspired by research data obtained for various components of Yoga (described in Supplementary Material). This has been done based on both ancient and modern literature. AYUSH protocol is the Government-recommended Yoga protocol for all ages and gender. As it is practiced on International Day of Yoga, this 45-min protocol incorporates all aspects of *Ashtanga* Yoga.

We have examined its effect on physiological, biochemical, and neuro-cognitive parameters. This study has also attempted to investigate the effect of yogic practices on various age groups, gender and to examine if one month of yoga practice helps to improve the dyslipidemia condition. We have also sought to ascertain whether yoga practice can affect significant physiological and biochemical changes in naïve and experienced volunteers.

## Methods

This was a prospective trial comparing clinical and laboratory findings before and after 1 month of yoga based on CYP intervention. The study design was approved by the Institutional Ethical Committee of the Post-graduate Institute of Medical Education and Research, Chandigarh, India (INT/IEC/2016/2565).

### Participant Recruitment

Participants were recruited during the International Yoga Day (IYD)-2016 (from May 2016 to June 2016, and data was analyzed during the same period) program organized by the Ministry of AYUSH at the Panjab University, Chandigarh, India. There were three groups: *Group 1*: healthy participants who did not perform any yoga (Yoga-naïve); *Group 2*: comorbid group; *Group 3*: trainer participants without a history of any comorbidity. A healthy control group was determined by normal vital parameters duly assessed by clinical colleagues as well as by self-disclosure of participants regarding health status. Individuals who have performed yoga for at least 15 days were included in the respective groups.

The recruited participants had given informed written consent as per the ethical guidelines before being included in the study. Physiological and biochemical parameters of the yoga-naïve group (healthy), comorbid group, and trainer group were obtained before and after 30 days of yoga practice. Socio-demographic details of participants were obtained before participation in the study.

### Inclusion and Exclusion Criteria

The enrolled participants were divided in three groups, i.e., naïve, comorbid, and trainer. Both male (*n* = 34) and female (*n* = 52) subjects in good health, with an age range of 18–55 years, and who had not performed yogic practices in the past 6 months were included in the “naïve group” (Table 1). Subjects with ongoing medications and/or had a prior history of surgery and cardiac problems were excluded from the naïve group and designated as the comorbid group. The “trainer group” consisted of the participants who were performing yogic practices at least from the past 6 months and more.

**Table 1:** Demographic characteristics of naïve and trainer groups along with the details of the experience of yoga practice.

**Parameters**	**Naïve**	**Trainer**
Gender (n)	Male = 34 Female = 52	Male = 54 Female = 27
Mean age (in years)	Male = 33.41 ± 9.9 Female = 26.89 ± 8.4	Male = 45.86 ± 15.28 Female = 34.83 ± 11.95
Duration of yoga practice (in years)	No experience	Male ~ 9 years Female ~ 11 years
Mean of yoga practice (male and female)		~ 9 years

### Intervention

Participants performed the CYP, which included components of asana, Pranayama, and stationary meditation standardized by the Ministry of AYUSH, Government of India, in consultation with experts (Supplementary Table S1). The duration of practice was 30 consecutive days with a single session in the morning between 6.00 and 7.00 a.m., all recruited participants were trained at least for 3 days to the above-mentioned protocol duly supervised by experienced practitioners. The experienced practitioners were trained for a minimum of 30 days under the program conducted by the Ministry of AYUSH before starting the yoga camp. The study participants were instructed for overnight fasting (after 10 p.m.) before the two time points with blood drawn (0 and 30 days during the CYP intervention) at baseline and after 30 days. Serum was analyzed for lipid profiles and fasting glucose.

### Physiological Parameters

Physical parameters, including weight, height, blood pressure, and pO_2_ levels were recorded at 5:30 a.m.. All the parameters were measured before the initiation of CYP intervention.

### Serum Extraction

About 3 ml blood was drawn by authorized and trained technicians from the antecubital vein at both the beginning and after 1 month of the study for biochemical investigations. The blood was drawn in a plain BD vacutainer at a fixed time (5:30 a.m.). The collected blood samples were subjected to centrifugation at 2,500 rpm for 20 min and an upper layer (i.e., serum) was collected in the vial for further biochemical investigations.

### Biochemical Analysis

Fully-automated clinical chemistry analyzer (Spectra Pro M) was used for biochemical analysis. Investigation of triglycerides (TG), total cholesterol (TC), high-density lipoprotein (HDL), low-density lipoprotein (LDL), and very low-density lipoprotein (VLDL) levels were carried out as per the manufacturer's instructions.

### Neurocognitive Analysis of Naïve Participants

For attention, Digit Span (DS) Test was performed. This test is a subtest of Wechsler Memory Scale (WMS)-III India, which is a measure of auditory attention and working memory. It includes two components, DS forward (DSF) and DS backward (DSB) tests. In the DSF test, the individual was presented with a series of digits and was asked to repeat them in the same order. In the DSB test, an individual was asked to repeat the digits in the reverse order of presentation. The score was based on the maximum number of digits recalled correctly. Similarly, a six-letter cancellation test (SLCT) was also administered for measuring attention. This test comprised of a sheet containing six target letters to be canceled. A working section comprised of 22 rows and 14 columns of randomly arranged letters that participants were asked to cancel as many as possible, at least six target letters in 90 s. To assess the executive functions, the Trail Making Test-A and Test-B were performed (TMT A and B). Part A dealt with visual attention. In this part, the subject was asked to connect a set of 25 circles with numbers (1-2-3) as quickly as possible while maintaining accuracy, on the other hand, part B measured cognitive flexibility and task switching. It contained both numbers and alphabets which had to be connected (1 a 2 b 3 c) as quickly as possible. The time needed to complete each test was noted at the end 26. For information processing speed, the coding test, which is also known as the digit symbol substitution test (DSST) from Wechsler's Adult Intelligent Scale, was performed. It is a test of visuomotor coordination, motor persistence, sustained attention, and responds to speed. This test consists of a sheet in which the numbers 1–9 are randomly arranged in 7 rows of 20 squares each. The subject was instructed to substitute a symbol for a corresponding digit with the given key. Scoring was as per the time taken to complete the task and errors were also noted.

### Blinding and Quality Assurance

The raw data and attendance of trainers, trainees, and staff were recorded in notebooks and digitized in Excel spreadsheets. These data were also validated independently to reduce bias and to preserve individuals' privacy. Immediately after collection, all blood samples were blinded with a master code and transported to a testing facility. The trainers documented participants' attendance and participation, and the protocol was video-recorded and/or still photographed daily. The trainer-to-participant ratio was 1:5.

### Statistical Analysis

Statistical Package for the Social Sciences (SPSS) version 16 was used for statistical analysis. The normality of data was determined by using the Kolmogorov–Smirnov test and an appropriate test was employed based on the normality of data. Paired *t*-tests and Wilcoxon signed ranks tests were utilized based on the distribution. *Post-hoc* analysis was also performed to measure the changes after 1 month of CYP in various groups, including trainer groups. Pearson's correlation analysis and scatter-plot matrix (through R stat) were performed to analyze the correlation of age and yoga experience with both anthropometric and biochemical parameters. The details of the dietary profile could not be obtained and hence did not correlate with the lipid profile of the participants. We have compared the baseline parameters of naïve participants with themselves (as they were not having a prior history of any exercise or yoga practice) after yoga intervention. Importantly, the naïve participant's data were further compared with comorbid and yoga practitioners' groups to understand the response of yogic practices to different physiological conditions and also to define the period of yoga exposure to bring homeostatic changes. The statistical significance threshold was set at *p* < 0.05.

## Results

### Demographic Details

**Figure 1:**
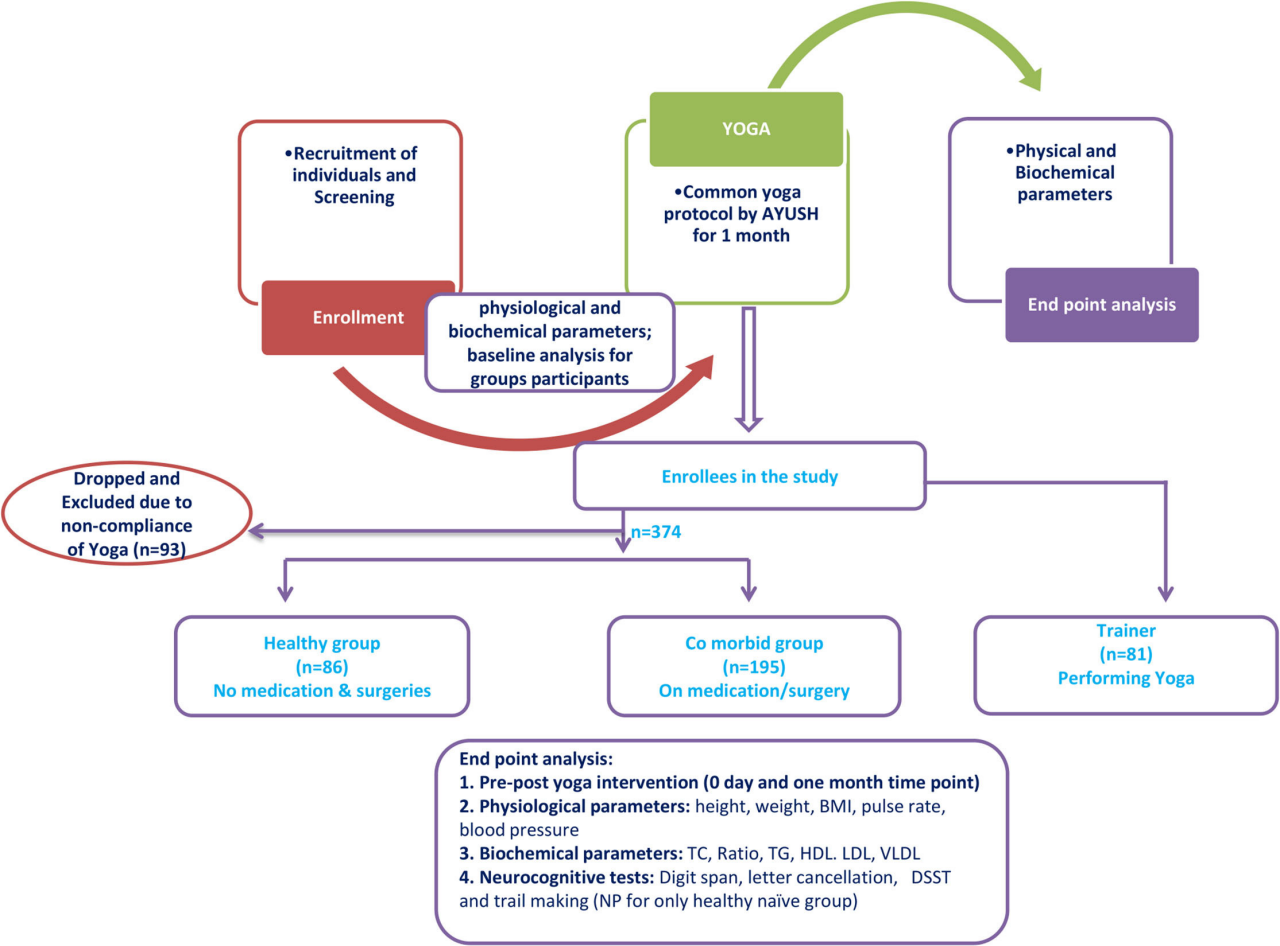
Plan of the study in a flowchart form.

A total of 374 participants were recruited for the study. Of these participants, 86 were assigned to the “healthy” yoga-naïve group. After the exclusion of 195 comorbid participants, 179 participants were found to be fit for the naïve group. However, 93 participants (out of 179) were excluded based on their compliance during the camp (minimum 15 days of yoga practice). Hence, the final analysis was carried out on 86 naïve participants. Additionally, 81 yoga trainers were also enrolled for comparison. Figure 1 describes the study design and demographic data are recorded in Table 1. The experience of the trainer group ranged from a minimum of 6 months to a maximum of 35 years. The demographic details of the trainer group are reproduced in Table 1. All participants maintained an average attendance for 21 days (ranging from 15 to 28 days) during the camp. It was made possible by continuous tracking, recalling, and attendance. No adverse events were reported during the study.

### Anthropometric and Biochemical Analysis in Healthy, Yoga-Naïve Participants Before and After 1 Month of Practice

**Figure 2:**
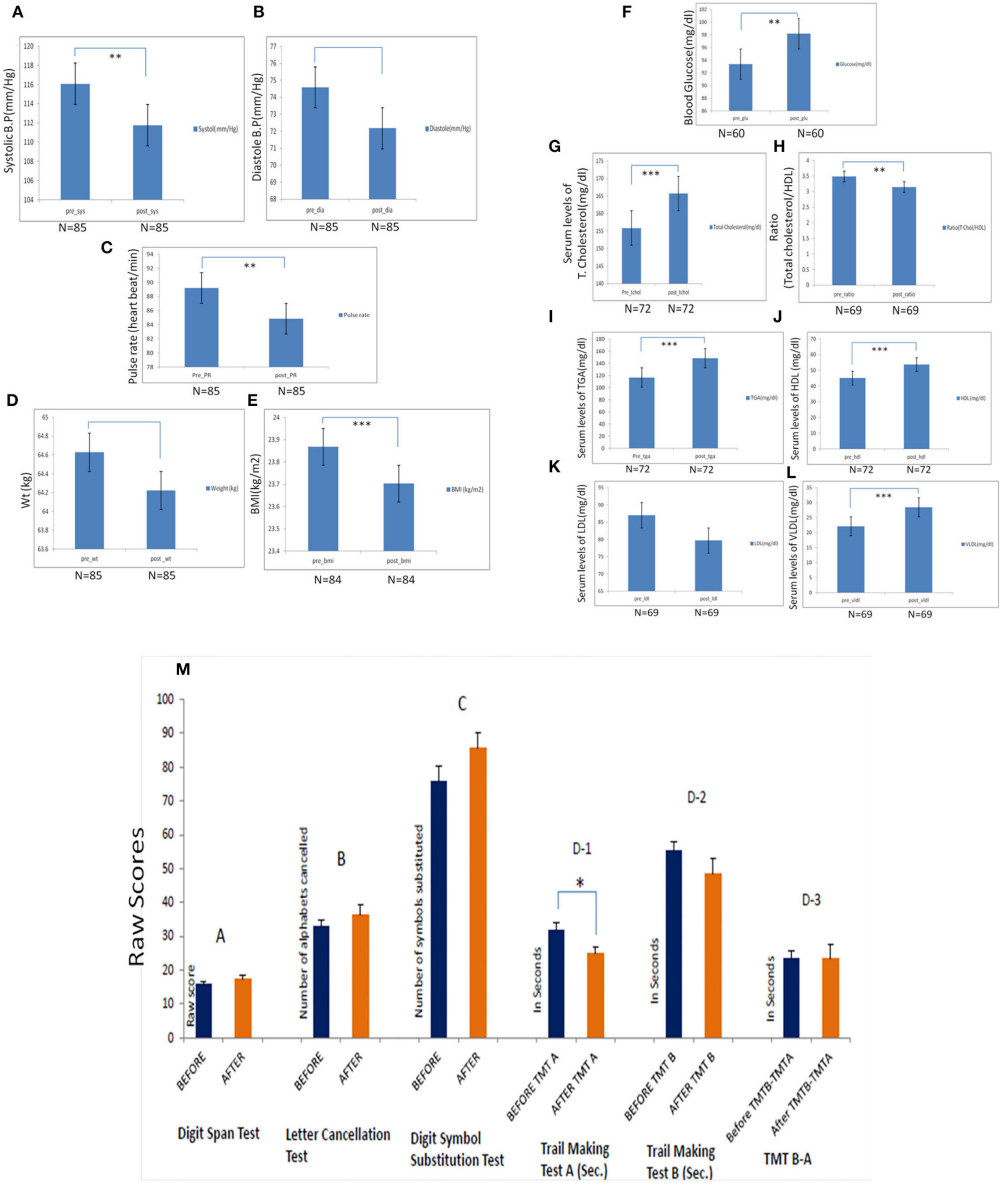
Pre- and post-analysis of naïve participants by estimation of physiological parameters after the 1 month of yoga-based Common Yoga Protocol (CYP). **(A)** Significant changes in the systolic blood pressure following 1 month of the camp (*p* = 0.001). **(B)** Changes in the diastolic blood pressure following 1 month of the camp (*p* = 0.013). **(D)** Changes in weight following 1 month of the camp. **(E)** Changes in body mass index following 1 month of the camp (*p* < 0.0001). Pre- and post-analysis of biochemical parameters after 1 month of the camp in naïve participants. **(F)** Pre- and post-serum levels of TG (mg/dl) total cholesterol (mg/dl) (*p* = 0.0001). **(G-I)** Pre- and post-serum and post-serum levels of LDL, HDL, and VLDL (mg/dl). **(J)** Pre- and post-serum and post-ratio (total cholesterol/HDL) (mg/dl) (*p* = 0.007). **(K,L)** Pre- and post-fasting blood glucose levels (mg/dl) (*p* = 0.012) of mindfulness-naïve individuals. **(M)** Neurocognitive profile of yoga-naïve individuals. The changes in neurocognitive profile before and after CYP at IYD 2016 on naïve-yoga individual (*n* = 15) after 1 month (mean duration: 19 days) (A) Digit Span Test: Measure of verbal working memory. (B) Letter Cancellation Test: Improvement in attention, visual search, and mental speed (C) Digit Symbol Substitution Test: A measure of executive functioning (D) Trail Making Test: A measure of visual attention and task switching. TMT A (D-1): A measure of cognitive processing speed significantly improved. TMT B (D-2): Measure of task switching improved. The Digit Span test here represents the combined score of Digit Span forward and backward. A: Digit span test, B: Letter cancellation test, C: Digit Symbol substitution test, D-1: Trial making test-A (TMTA), D-2: Trial making test-B (TMTB), D-3: TMTA-TMTB. Bar is showing standard error mean (SEM). ^*^Statistical significance ≤ 0.05, ^**^ Statistical significance 0.001 and ^***^ Statistical significance <0.0001. Bar is showing standard error mean (SEM).

Comparing pre- and post-levels after 1 month of practice, the yoga-naïve, and healthy participant group demonstrated a significant decrease in systolic blood pressure, pulse rate, and BMI (Figures 2A,C,E). There was also a trend toward decreased diastolic blood pressure, but this did not reach statistical significance (Figures 2A,D). Laboratory findings were within the normal range in the healthy group. A statistically significant decrease in TC/HDL ratio and a statistically significant increase in serum HDL were observed in this group. Serum LDL was reduced in healthy participants but did not reach statistical significance (Figures 2F–L). We did not find significant alteration in systolic pressure in naive-healthy participants after one month yogic practices (Figure 2B).

### Comparative Post-CYP Analysis of Anthropometric and Biochemical Parameters in Naïve Healthy Compared With Experienced Practitioners

**Figure 3:**
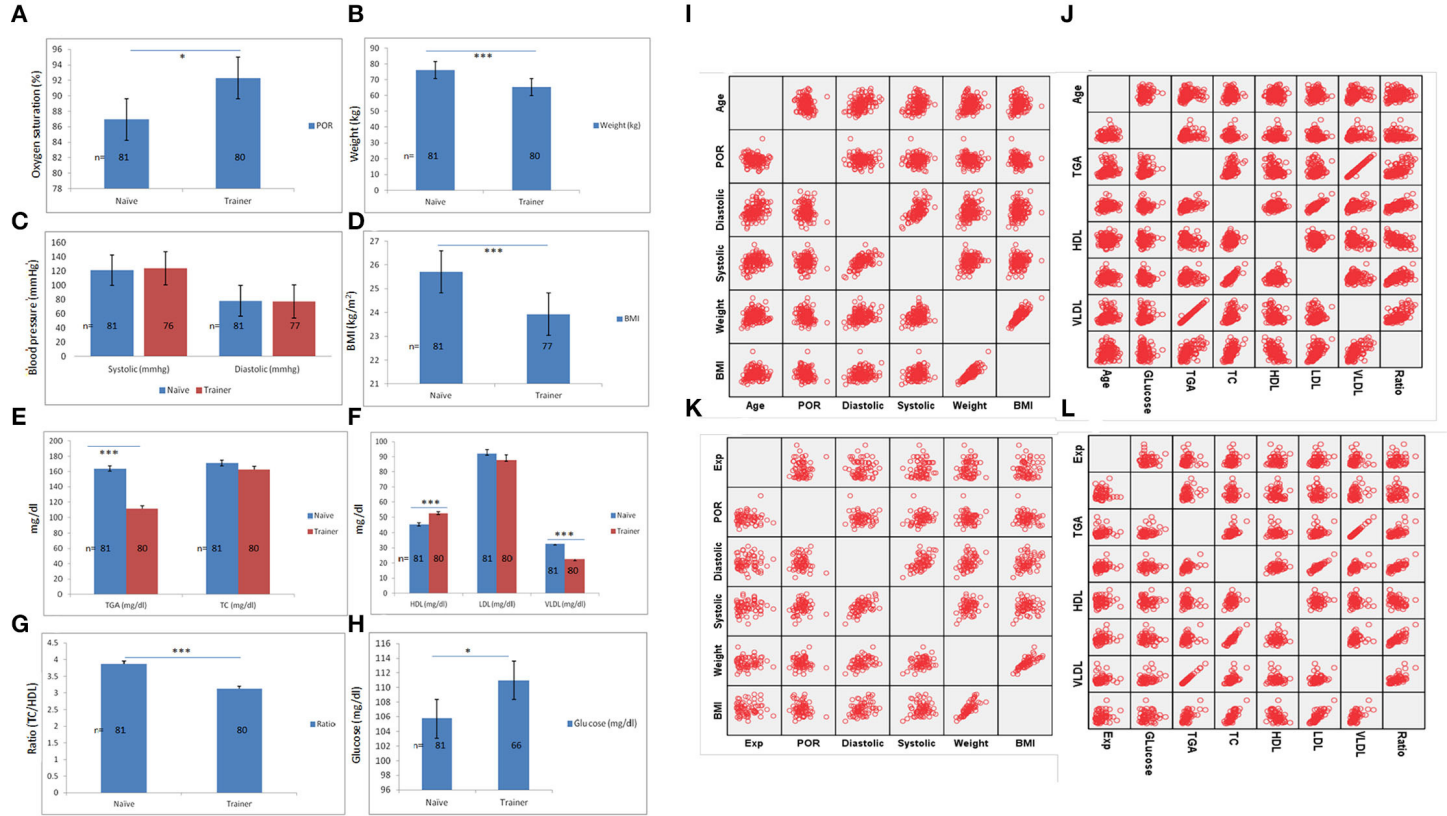
Physiological-biochemical measurements and comparison between naïve and trainer groups. Various physiological parameters of naïve participants before yoga exposure including **(A)** POR, **(B)** weight, **(C)** blood pressure (systolic and diastolic), and **(D)** BMI and its comparison with trainer groups. Biochemical analysis of same naïve participants for **(E)** TG and TC (mg/dl), **(F)** HDL, LDL, and VLDL (mg/dl), **(G)** ratio (TC/HDL), and **(H)** serum glucose levels (mg/dl) and further compared with trainer group shown significant alteration in various important metabolic pathways. **(I,J)** The scatter-plot matrix is showing the correlation of age with physiological and biochemical parameters analyzed by bivariate correlation analysis. **(K,L)** Scatter-plot matrix is showing the correlation of yoga experience on various anthropometric and biochemical values. ^*^Statistical significance ≤ 0.05, ^**^statistical significance 0.001 and ^***^statistical significance <0.0001. Bar is showing standard error mean (SEM).

Anthropometric and lab testing were performed on trainers on day 30. Controlling for age differences between yoga-naïve and trainer groups at day 30, the trainer's group had significantly higher percent oxygen saturation and lower BMI as compared to the yoga-naïve group (Figures 3A,B,D). However, diastolic and systolic pressure of both groups (naive and trainers) were not found be to significantly altered (Figure 3C). The trainer group also had significantly lower LDL and TC/HDL ratios and higher HDL levels compared with the yoga-naïve group (Figures 3E–H).

To estimate a temporal correlation between age and yoga experience, we analyzed the effect of age with physiological and biochemical parameters by scatter-plot matrix. This showed a significant positive correlation of age with blood pressure, BMI, TG, TC, and VLDL (Figures 3I,J; Supplementary Table S4). Pearson's analysis of yoga experience with physiological parameters did not show any significant correlation between yoga training with anthropometric values. Except for yoga experience, the correlation between other parameters was found to be significant. TC and LDL values both had a significant positive correlation with yoga experience in the participants (Supplementary Figures S3K,L; Supplementary Table S5).

Analysis based on various age groups in naïve and trainer participants has also revealed significant alteration parameters of physiological (e.g., weight, BMI, percent oxygen saturation, and systolic pressure) as well as lipid metabolism (including TG, HDL, LDL, VLDL, and ratio). Results suggest that adoption of these practices could show a better effect after the age of >30 years (Supplementary Table S2).

Moreover, gender-based analysis in naïve and trainer participants also showed alterations in various physiological (including weight, BMI, systolic, and diastolic pressure) and biochemical parameters (e.g., levels of TG, HDL, VLDL, and ratio) which suggest a differential effect of these practices on male and female participants (Supplementary Table S3).

### Neurocognitive Analysis in Naïve Individuals Performing Yoga

There was a significant reduction in the Trail making test-A to assess psychomotor speed (*p* = 0.015) in the naïve participants after the yoga duration of 1 month. This result indicated an improvement in the psychomotor speed. Improvements in the TMT B test results were also observed though statistically non-significant. Improvements in tests related to attention and information processing speed (SLCT, DST, and DSST) were also observed after performing a 1-month yoga protocol (Figure 2M).

### Duration of Yoga Practices in Trainers

The trainer group was categorized into three sub-categories based on the length of their period of yoga practice (yoga experience, Table 1) to evaluate the probable effective period of yoga: (i) up to 3 years; (ii) 3–10 years; and (iii) >10 years. Physiological parameters such as weight and BMI were significantly lower and oxygen saturation was significantly higher in trainers who had been practicing yoga for over 3 years (Supplementary Figures S1A–C; Table 2). No difference in systolic and diastolic blood pressure was noticed among any of the groups (Supplementary Figure S1D; Table 2). TG and VLDL were significantly lower and HDL was significantly higher in trainers with a longer duration of yoga practice. LDL and TC were lower in trainers with a longer duration of yoga practice, but this did not show any statistical significance (Supplementary Figures S1E–H; Table 2).

**Table 2:** Comparative analysis (means with standard deviation) of physiological and biochemical factors in different groups based on their experience in yogic practices with naïve participants.

**Parameters**		**Mean ±SD**				***p-value***					
		**Naïve (*n* = 81)**	**Upto 3 years (*n* = 24)**	**>3–10 years (*n* = 27)**	**>10 years (*n* = 28)**	**Naïve vs. <3 years**	**Naïve vs. 3-10 years**	**Naïve vs. > 10 years**	**< 3 vs. 3-10 years**	**< 3 vs. >10 years**	**3–10 vs. >10 years**
Physiological	Age	42.88 ± 9.84	41.54 ± 13.72	38.15 ± 15.14	46.39 ± 9.73	0.969	0.334	0.584	0.775	0.514	0.074
	Weight	75.99 ± 14.10	67.00 ± 13.29	61.44 ± 11.30	68.00 ± 9.52	0.031	<0.0001	0.049	0.50	0.994	0.314
	BMI	27.14 ± 4.97	24.86 ± 4.75	22.49 ± 4.45	24.56 ± 4.22	0.234	0.001	0.116	0.385	0.997	0.489
	POR (%)	86.94 ± 12.74	89.00 ± 14.15	94.52 ± 13.47	92.96 ± 17.49	0.940	0.119	0.282	0.579	0.793	0.982
	Systolic blood pressure (mmhg)	121.17 ± 12.36	123.52 ± 10.31	122.89 ± 15.09	123.69 ± 13.66	0.867	0.999	0.977	0.947	0.774	0.973
	Diastolic blood pressure (mmhg)	77.94 ± 10.06	75.86 ± 9.99	77.63 ± 11.21	78.96 ± 9.34	0.896	0.948	0.859	0.999	1.0.	0.997
Biochemical	TG (mg/dl)	163.94 ± 84.06	108.13 ± 69.25	102.48 ± 52.06	124.50 ± 44.15	0.012	0.003	0.102	0.994	0.878	0.012
	TC (mg/dl)	171.19 ± 32.31	155.54 ± 30.60	156.96 ± 35.91	174.68 ± 36.83	0.162	0.306	0.973	0.999	0.224	0.283
	HDL (mg/dl)	45.51 ± 9.08	51.96 ± 10.72	52.89 ± 7.08	52.68 ± 8.32	0.024	0.004	0.005	0.987	0.994	1.000
	LDL (mg/dl)	91.99 ± 24	81.33 ± 24.23	83.70 ± 31.77	97.21 ± 30.28	0.410	0.591	0.853	0.992	0.217	0.330
	VLDL (mg/dl)	32.81 ± 16.85	22.46 ± 13.81	20.37 ± 10.45	25.00 ± 8.96	0.024	0.002	0.108	0.966	0.939	0.699
	Ratio (TC to HDL)	3.87 ± 0.81	3.04 ± 0.74	2.98 ± 0.74	3.34 ± 0.75	<0.0001	<0.0001	0.026	0.993	0.589	0.392
	Fasting blood glucose (mg/dl)	102.23 ± 22.92	110.56 ± 23.79	107.80 ± 27.28	114.78 ± 26.33	0.633	0.802	0.195	0.987	0.959	0.805

*TG, triglycerides; TC, total cholesterol; HLD, high density lipoprotein; LDL, low-density lipoprotein; LDL, very low-density lipoprotein*.

### The Effect of the AYUSH Yoga Protocol on the Participants With Medical Comorbidities

A total of 195 participants out of 374 had at least one medical or surgical problem in their recent history. In this group, pulse rate was significantly increased after the 1-month yoga practice (Table 3) along with a significant reduction in weight of the participants. A significant enhancement in TG, HDL, and VLDL was observed in the comorbid group tantamount to the corresponding LDL and ratio (total cholesterol to HDL) alteration after 1 month of AYUSH yoga protocol. Additionally, blood fasting glucose levels were significantly increased (Table 3) and found to be similar to our remaining results.

**Table 3:** Comparative analysis of both physiological and biochemical parameters in comorbid participants after 1 month of AYUSH yoga protocol (*n* = 119).

**Parameters**	**Pre** **(mean ±SD)**	**Post** **(mean ±SD)**	**Mean difference**	***t*** **-value**	***p*** **-value**
Triglycerides (mg/dl)	131.42 ± 72.93	146.89 ± 77.13	−15.47	−2.79	0.006
Total cholesterol (mg/dl)	173.33 ± 144.08	169.71 ± 35.13	3.62	0.27	0.79
HDL (mg/dl)	47.04 ± 10.04	55.86 ± 12.92	−8.82	−9.64	<0.0001
LDL (mg/dl)	87.46 ± 24.27	84.09 ± 26.87	3.37	1.83	0.07
VLDL (mg/dl)	25.43 ± 13.23	30.54 ± 20.09	−5.11	−2.74	0.007
Ratio	3.49 ± 0.87	3.12 ± 0.88	0.37	5.98	<0.0001
Fasting Blood glucose (mg/dl)	108.22 ± 31.36	125.45 ± 56.68	−17.23	−3.31	0.001
Pulse rate (per min)	87.67 ± 11.29	83.17 ± 13.40	4.50	3.06	0.003
Diastolic blood pressure (mmhg)	75.55 ± 9.94	74.64 ± 9.08	0.909	0.775	0.440
Systolic blood pressure (mmhg)	118.33 ± 14.69	117.41 ± 13.08	0.920	0.689	0.492
Weight (kg)	70.18 ± 14.57	69.66 ± 14.36	0.53	1.36	0.18

### Comparison of Physiological-Biochemical Parameters Between the Healthy vs. Comorbid Participants

**Figure 4:**
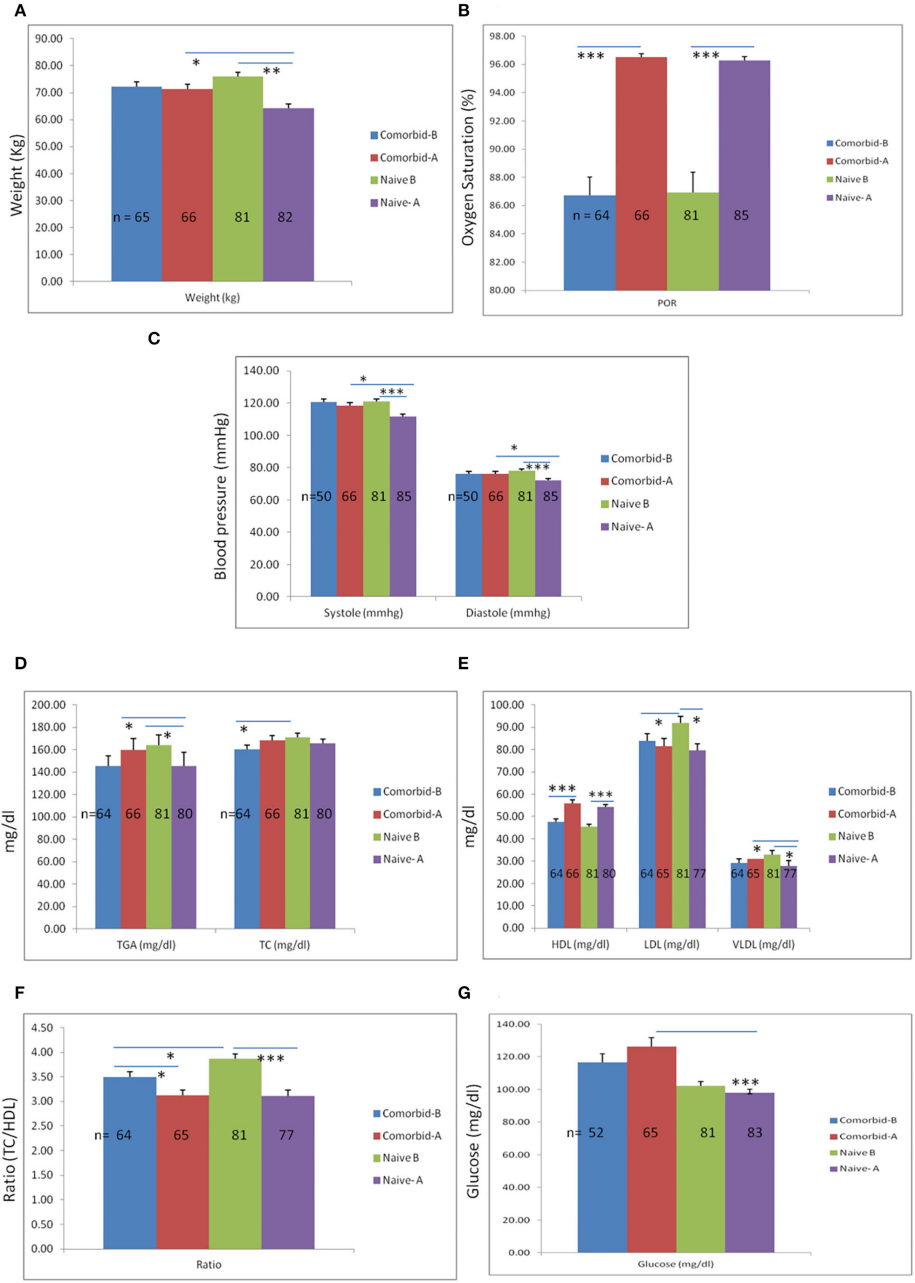
Comparison of physiological-biochemical parameters between comorbid and naïve participants. A month of yoga practice in both groups has demonstrated a significant weight reduction. **(A)** However, weight reduction was significantly more in the naïve group as compared to the comorbid group. Additionally, significant reductions in blood pressure (systolic and diastolic) were observed in the naïve group after a 1-month yogic practice, although reductions in systolic and diastolic blood pressure in the naïve participants were more as compared to the comorbid group. **(C)** Percent O_2_ saturation rate was significantly altered after 1 month of yoga practice in both groups. **(B)** Results of biochemical analysis have shown a significant reduction in TG levels in naïve participants after 1 month of yoga practice and increased levels of TG in the comorbid group after 1 month. Additionally, no significant changes were observed in TC levels except base levels (of TC) between comorbid and naïve groups before the yoga intervention. **(D)** However, HDL levels were significantly higher in both groups after performing yoga intervention for 1 month. **(E)** Similarly, a significant reduction in LDL levels was also observed in both groups after 1 month of yoga practice. **(E)** However, a significant reduction in VLDL was only shown in the naïve group but did not show significant changes in the comorbid group. **(F)** Moreover, the overall ratio of TC to HDL was reduced significantly in both groups, though the base level ratio was significantly higher in naïve participants as compared to comorbid participants. **(G)** No significant differences were observed in glucose levels in any groups except the comorbid and naïve groups after 1 month of yoga intervention. ^*^Statistical significance ≤ 0.05, ^**^statistical significance 0.001 and ^***^statistical significance <0.0001. Bar is showing standard error mean (SEM).

While both groups improved, greater improvements in post-yoga practice were seen in the healthy group compared with the comorbid group. Significant differences were found after a month of practice in physiological parameters such as systolic blood pressure (*p* = 0.004), diastolic blood pressure (*p* = 0.013), and weight (*p* = 0.003) when the comorbid yoga group and healthy yoga group were compared, signifying the effectiveness of yoga in healthy participants as compared to the comorbid group. However, a significant increase in percent oxygen saturation (POR) was observed in both healthy and comorbid groups (Figures 4A–C). The analysis of biochemical parameters has revealed that revealed that glucose (*p* ≤ 0.001), TG (*p* = 0.030), and VLDL (*p* = 0.026) showed significant differences between healthy and comorbid groups after a 1-month of practice (Figures 4D,E,G, respectively). Notably, pre- and post-intervention comparison of participants revealed significant differences in O_2_ saturation (*p* ≤ 0.001), systolic blood pressure (*p* ≤ 0.001), diastolic blood pressure *p* ≤ 0.001), weight (*p* ≤ 0.001), TG (*p* = 0.018), HDL (*p* ≤ 0.001), LDL (*p* = 0.002), VLDL (*p* = 0.009), ratio (*p* ≤ 0.001), but pre- and post-intervention comparison of the comorbid group revealed significant differences only in O_2_ saturation (*p* ≤ 0.001) and HDL (*p* ≤ 0.001), TC/HDL ratios (*p* = 0.016) (Figures 4A–F).

## Discussion

Stress which is an inevitable aspect of life is a major contributor to the development of several ailments including coronary heart disease (CHD). An investigation performed on cardiac patients revealed that the patient number visiting the clinics reduced to a much greater extent when they practiced yoga (24). Yoga and Pranayama have varied health benefits starting from decreasing stress through the hypothalamus-pituitary-adrenal axis (HPA) which is a major risk factor for T2DM and CVDs. Its effects are present and seen in various other diseases such as hypertension *via* the parasympathetic activation of the vagal nerve, reducing coronary atherosclerosis with an improvement in the lipid profile, cholesterol levels, and body weight. Yoga has its effects on cardio-respiratory efficiency and physical fitness (25). For cardiovascular health, yoga has been shown to display neuroendocrine regulation with sympathetic tone reduction and an increase in parasympathetic response resulting in an increase in exercise capacity, and reducing the IL6 level markers. It also directly affects the sub-clinical and clinical outcomes with a reduction in heart rate, an increase in HDL levels and reduction of LDL levels, a decrease of atherosclerotic lesions, number of arrhythmia episodes generating the need for revascularization (26). A study performed on 30 medical students aged 18–21 in the year 2016, who were administered *Sudarshan Kriya* Yoga, a variant of Pranayama (breathing aspect of Yoga), showed significant improvement in the mean arterial blood pressure, blood glucose, and cholesterol after 3 months of practice (27). These studies indicate that yoga practice may modify blood-related parameters and thereby contribute to the prevention of CVD and other metabolic disorders. Hyperlipidemia is also a risk factor for neurodegenerative diseases such as Alzheimer's disease (28–30). Several studies have reported cognitive enhancement from yoga practice (31–33). This has been often attributed to neurological underpinnings and blood rheological parameters affected by blood pressure and lipid contents. Our results of neurocognitive data have also suggested the statistically enhanced psychomotor performance in na participants after one month of CYP practice. the na participants showed better response to questions related to attention and information processing as an outcome of one month IYD protocol.

The importance of lipid profile in health is evident from past studies which have shown that hyperlipidemia is a risk factor for neurodegenerative diseases, e.g., Alzheimer's disease in which cognitive decline is observed (29, 34). Several studies have even reported cognitive enhancement among individuals who practiced yoga. However, as different forms of yoga from varying yoga schools have been used in various studies, it is imperative that standardized practices are used for beneficial effects by using a national consensus Common Yoga Protocol (CYP) or the AYUSH protocol (35, 36). A study by Sharma et al. has shown the effect of slow Pranayama (*Nadishodhana, Pranav*, and *Savitrikapalabhati*) and fast Pranayama which included *Bhastrika* and *Kukkuriya* on the cognitive function of the healthy individuals. Both aspects have been covered in the yoga-based CYP. Results of inter-group comparison have shown that both types of Pranayama are beneficial for cognitive function, but a rapid Pranayama technique has additional effects on the executive function of manipulation in auditory working memory, central neural processing, and sensory-motor performance (31).

Studies have shown that the latter may ameliorate cholesterol levels, blood pressure, stress, type 2 diabetes, and other risk factors associated with CHD (37–40). Anderson et al. performed a meta-analysis and observed the effect of transcendental meditation on blood pressure and found a positive effect on these patients suggesting an overall reduced fatality due to stroke and CHD (41–44). Furthermore, there are several lines of evidence that indicate yoga is a safe and inexpensive treatment for cardiovascular diseases. However, it is crucial to set a standardized and validated yoga protocol to corroborate its influence on people suffering from CVDs (45). Most of the previous studies have examined only one component or school of yoga or evolved as such without any Delphi protocol of discussions. This may or may not be acceptable to all, as there could be commercial interests in it. The AYUSH protocol is the “Government-mandated Yoga protocol” or a standardized yoga protocol and has more acceptability among the masses than yoga protocols available in different Yoga schools. A few of these protocols are not even mentioned in Yoga literature or *shlokas*. The validation and safety of the AYUSH protocol are imperative from both public health intervention perspectives as well as scientific analysis. It is practiced by millions of individuals on the International Day of Yoga as it is mandated by the Government of India. Also, there is no comparative study to demonstrate the safety and efficacy of AYUSH. This is possible by analyzing the various parameters between short-term and long-term practitioners.

Our observation of a significant decrease in pulse rate (heartbeat/min) and blood pressure after 1 month of CYP practice is consistent with previous studies that indicate yoga practice lowers blood pressure (46–48). Decreased systolic and diastolic blood pressure, and reduction in HR are effects of para-sympathomimetic effects of yoga interventions (49, 50). This study did not find any significant difference in systolic-diastolic BP in the comorbid group as compared to the naïve groups. Though the reason could not be ascertained but decreased vasodilatory effects due to decreased nitric oxide in diseased endothelium could be a reason. Earlier studies by Chauhan et al. (51) among others had reported similar effects of 1-month yoga and meditation practice in the reduction of systolic-diastolic BP and BMI.

CYP can be argued as a preventive and protective measure for a developing country like India whose health budgets are fast escalating. POR has been found to have a significant difference in both the groups, even though another arm of the sham or general exercise group was not available. In this study, we report increased POR in practitioners which is concomitant with the increase in blood glucose levels in most yoga practice groups. Similarly, triglycerides and VLDL levels were also increased after one month of yogic practices. Though, the levels were not go beyond the recommended values for both biomolecules. After yoga practices, both results have indicated that aerobic conditions can increase the cellular metabolic processes and also maintenance of pH of the body by regulating the respiratory processes. Mindfulness rejuvenating effect is ascribed to breathing exercises centered around forced breathing.

In this study, we report an increased percentage of saturation of oxygen in yoga practitioners. Our study showed statistically significant improvement in TC/HDL ratios. High TC/HDL ratios are correlated with the risk of heart disease. A study by Balaji et al. (52) and Tang et al. (53) also demonstrated improved TC/HDL ratios in yoga practitioners and attributed this to increased intracellular levels of hepatic lipase, lipoprotein lipase metabolism, and a successive increase in uptake of triglycerides by adipose tissue. Similarly, 12 weeks of yoga practice were found to reduce fasting blood glucose levels and improve lipid profiles in polycystic ovary syndrome (PCOS) (54, 55). To our knowledge since there are varying types of yoga practices, it is difficult or indeed impossible to delineate the benefits. This to our knowledge is the first reported study in the world of the benefits of a standardized yoga protocol.

## Limitations

Our study was limited by low numbers of healthy yoga-naïve participants. Therefore, there is a high risk of type one error, in that, a statistical significance of some outcomes might have been seen with higher numbers of participants. Another limitation of our study was that it was a short-term study and was not randomized. Also, the healthy group was comprised of people aged 18–55, while the comorbid group was comprised of a heterogeneous group including healthy individuals over age 55 and younger individuals with medical issues. This did not allow us to separately analyze the effect of the CYP on a healthy older population.

We have not undertaken any classification as reported in references (46, 47) and every individual was treated the same. If that introduced any bias or not is unclear as more scientific research is mandated to understand these subsets in the context of modern medicine. The milder effect of the yoga-based CYP on the comorbid group suggests the need for varying protocols, since recovery periods were found to be slow in the comorbid group. Finally, large multicentric randomized trials are required to confirm these findings. However, as yoga is an inexpensive and easy technique, it can be recommended for the primary prevention of CVD (56).

## Conclusion

Our results showed that Common Yoga Protocol improved the key metabolic and physiological processes in the yoga intervention group. Healthy yoga-naïve participants showed greater improvement than yoga-naïve participants with comorbidities. The results from these comparisons indicate that yoga is useful for maintenance of good health parameters. However, the length of the study was shorter and we were not able to evaluate if the effects last after leaving yoga practice. Whether CYP holds any potential effect in the treatment of hyperlipidemia can only be confirmed by a randomized control study.

## Data Availability Statement

The original contributions presented in the study are included in the article/Supplementary Material, further inquiries can be directed to the corresponding author/s.

## Ethics Statement

The studies involving human participants were reviewed and approved by Institutional Ethical Committee of the Post-graduate Institute of Medical Education and Research, Chandigarh, India (INT/IEC/2016/2565). The patients/participants provided their written informed consent to participate in this study.

## Author Contributions

AA: conceptualization and principal investigator. KS, NS, and RT: collection of sample, data and writing of the initial manuscript, and analysis of data. IB-R, AV, DK, MS, and VP: writing, copy editing, and repeated revision based on data. SB, SM, PB, PK, and VB: collection of samples and data and sample processing. AG, DP, KJ, VGa, BM, NT, and AC: compilation and electronic entries of the data. NK and PM: assistance in yoga follow up. KB, VP, and AB: data from medical parameters. AB, MM, GS, and SS: assistance in conceptualization of study design. VGu: assistance in providing the medical staff and equipments. NM, RM, and RKu: yoga training to the volunteers and arrangement of the space and other accessories for yoga participants. DM: writing and revision of the manuscript. MM and HA: neuropsychology analysis of the volunteers. RKa provided chemicals used in sample processing. SS: statistical analysis of the data. All authors contributed to the article and approved the submitted version.

## Conflict of Interest

The authors declare that the research was conducted in the absence of any commercial or financial relationships that could be construed as a potential conflict of interest.

## Publisher's Note

All claims expressed in this article are solely those of the authors and do not necessarily represent those of their affiliated organizations, or those of the publisher, the editors and the reviewers. Any product that may be evaluated in this article, or claim that may be made by its manufacturer, is not guaranteed or endorsed by the publisher.
