# Supplementary material for: Yoga as a Preventive Intervention for Cardiovascular Diseases and Associated Comorbidities: Open-Label Single Arm Study
Source: Front Public Health. 2022 Jun 13;10:843134. doi: 10.3389/fpubh.2022.843134 (PMC9234218; doi:10.3389/fpubh.2022.843134)
Supplement: Supplementary file 1 [file Data_Sheet_1.docx]

**YOGA as a preventive intervention for Cardiovascular and neurocognitive diseases: open label single arm study**

**Supplementary File**

**Materials and Method**

**Recruitment of participants:**

The study resulted after a call from the Chandigarh administration to assemble for one month to practice Common Yoga Protocol with the Prime Minister on June 21, 2016. As a result, a large number of individuals gathered to practice Yoga for more than a month. This ensured their place in UNESCO Capitol Complex of Chandigarh, where the Mr Narender Modi performed Yoga along with the general public. The inclusion of participants was based on general health criteria, regularity of practice and compliance with yogic practices during the protocol implementation. Various deliberations and study designs were also discussed at the national conclave held in Chandigarh, organized by the Chandigarh Administration and co-organized by the Annals of Neurosciences, the official Journal of Indian Academy of Neurosciences. Participants performed the AYUSH yoga protocol for 1 month period under the guidance of trained yoga experts authorized by the Chandigarh administration. These experts performed yoga along with participants on an elevated stage erected at Panjab University Grounds. Before the start of the one month camp, trainers were given a three day orientation on the 45 minute protocol between 6 and 7 am with the help of experts from the Patanjali Foundation, Department of Sports, Panjab University and Vyakti Vikas Kendra, Bengaluru, under the overall supervision of the Honorable Dr. H.R. Nagendra, the Chancellor of SVYASA (an NAAC A+ rated Yoga University) and DC of Chandigarh Mr Joshi and the Joshi Foundation. These experts supervising the session had previous experience of teaching in schools, colleges and other Institutes for at least 6 months to 35 years. The event was performed in the open ground on yoga mats provided by Panjab University for each of the participants. These were laid out at 5 am by the employees of the University. Daily photographs, videos, and "WhatsApp" messages were continuously exchanged several times a day to keep the volunteers motivated and compliant to the daily attendance. The objectives of the study were explained to the subjects and both written and video consents, along with video feedbacks, were obtained from volunteers. The details of the standardized CYP are depicted in **Figure 1**. All the experimental records were maintained in a GLP format.

**Yoga Protocol**

The participants performed the protocol for 45 minutes under trainers’ daily supervision at the same time and place. This included prayer, *asanas*, *pranayma* and meditation. Details of the protocol recommended by the Ministry of AYUSH (Ayurveda, Yoga and Naturopathy, Unani, Siddha and Homoeopathy) are given in **Table S1**. This is also available in the public domain. The subjects were advised to follow their routine diet and exercise pattern during the period of study.

| **S. No.** | ***ASANAS*** | **PROTOCOL** | **DURATION** |
| --- | --- | --- | --- |
| 1. | Prayer | To enhance the benefits of practice. | 2 minutes |
| 2. | Loosening practices | Increases microcirculation:   - Neck bending - Trunk movement   (*Katishaktivikasak*)   - Knee movement | 5 minutes |
| 3. | Yogasanas (1 minute per asana) | **Standing postures**   - *Tadasana*   (Palm tree pose)   - *Vkrsasana*   (Tree posture)   - *Padahastasana*   (The hands and feet posture)   - *Ardhacakrasana*   (The half wheel posture)   - *Trikonasana*   (The Triangle Posture)  **Sitting postures**   - *Bhadrasana*   (The firm auspicious posture)   - *Ardhaustrasana*   (The half camel posture)   - *Sasankasana*   (The Hare posture)   - *Ardhaustrasana* - *Vakrasana*   (The spinal twist posture)  **Prone postures**   - *Bhujangasana*   (The Cobra posture)   - *Salabhasana*   (The Locust Posture)   - *Makarasana*   (The Crocodile Posture)  **Supine postures**   - *Setubandhasana*   (The Bridge Posture)   - *Uttana padasna* - *Ardhahalasana*   (Half Plough posture)   - *Pavanamuktasana*   (The Wind releasing Posture)   - *Savasana*   (The Dead body Posture) | 15 minutes |
| 4. | Kapalbhati | Forceful exhalation by contracting the abdominal muscles. | 2 minutes |
| 5. | *Pranayama* (2 minutes each) | - *Nadishodhana* or *Anulomvilom*   (Alternate nostril breathing)   - *Satali Pranayama* - *Bhramari pranayama* - *Dhyan* in *Shambavi mudra* | 8 minutes |
| 6. | Meditation | For stress free deep relaxation and silencing of mind. | 12 minutes |
| 6. | *Sankalpa* | Commitment to be healthy, happy, peaceful and joyful human being. | 1 minute |
| 7. | *Shanti path* | Prayer for happiness, health and peace for all. | 1 minute |

**Table S1:** Summary of AYUSH Common Yoga Protocol 2016.

|  |  | Mean ± SD | | | | | | *P value* | | |
| --- | --- | --- | --- | --- | --- | --- | --- | --- | --- | --- |
| Parameters | **Variables** | **Naïve**  **(Grp A)**  **(n=16)** | **Trainer (Grp A)**  **(n=24)** | **Naive**  **(Grp B)**  **(n=46)** | **Trainer**  **(Grp B)**  **(n=36)** | **Naive**  **(Grp C)**  **(n=29)** | **Trainer**  **(Grp C)**  **(n=16)** | **Group A** | **Group B** | **Group C** |
| Physiological | Age | 29.69±1.66 | 26.58±3.81 | 42.08±5.54 | 42.19±4.49 | 57.50±3.41 | 59.58±3.81 | **0.001** | 0.923 | **0.04** |
|  | Weight | 66.44±11.54 | 61.75±13.78 | 77.07±14.22 | 67.47±11.27 | 81.50±13.40 | 61.75±13.78 | 0.252 | **0.001** | **<0.0001** |
|  | BMI | 23.89±2.62 | 23.14±5.07 | 28.17±5.43 | 24.49±4.25 | 26.63±3.84 | 23.14±5.07 | 0.551 | **0.001** | **0.016** |
|  | POR | 93.06±10.38 | 92.63±16.58 | 86.06±12.29 | 91.66±12.97 | 84.00±12.71 | 92.63±16.58 | 0.919 | **0.051** | 0.082 |
|  | Systole | 114.38±11.53 | 115.92±9.65 | 119.15±9.07 | 125.09±14.65 | 131.00±13.28 | 115.92±9.65 | 0.662 | **0.042** | 0.482 |
|  | Diastole | 72.63±10.65 | 73.52±10.57 | 78.63±10.19 | 79.41±10.09 | 81.43±6.99 | 73.52±10.57 | 0.797 | 0.839 | 0.453 |
| Biochemical | TG | 124.38±89.63 | 86.13±47.86 | 173.50±85.40 | 117.53±48.67 | 163.86±63.09 | 86.13±47.86 | 0.095 | **<0.001** | **0.054** |
|  | TC | 162.81±27.81 | 145.42±34.90 | 174.50±35.75 | 173.44±37.19 | 174.79±27.32 | 145.42±34.90 | 0.089 | 0.897 | 0.482 |
|  | HDL | 46.25±8.69 | 50.88±7.79 | 44.28±8.87 | 54.27±9.46 | 48.93±8.59 | 50.88±7.79 | 0.096 | **<0.001** | 0.193 |
|  | LDL | 91.69±17.00 | 76.54±29.78 | 93.97±25.87 | 95.88±30.81 | 93.00±28.48 | 76.54±29.78 | **0.048** | 0.766 | 0.944 |
|  | VLDL | 24.81±17.76 | 18.00±9.90 | 34.76±17.15 | 23.58±9.85 | 32.79±12.65 | 18.00±9.90 | 0.165 | **<0.0001** | **0.052** |
|  | Ratio | 3.60±0.85 | 2.87±0.74 | 3.97±.81 | 3.22±0.74 | 3.75±0.78 | 2.87±0.74 | **0.009** | **<0.0001** | **0.017** |
|  | Glucose | 92.50±8.21 | 106.24±24.04 | 103.28±25.60 | 111.90±25.02 | 104.29±18.57 | 106.24±24.04 | **0.022** | 0.064 | 0.584 |

**Table S2**: Mean (with SD) and statistical significance in various physiological and biochemical parameters between different groups based on their age range.

**Table S3**: Tabular representation of gender wise comparison of physiological and biochemical parameters before and after mindfulness exposure in naïve and trainer participants. FN: Female naïve; FT: Female trainer; MN: Male naïve; MT: male trainer.

|  |  | Mean ± SD | | | | *P value* | | | |
| --- | --- | --- | --- | --- | --- | --- | --- | --- | --- |
| Parameters |  | **Female Naïve**  **(n=37)** | **Female Trainer**  **(n=27)** | **Male Naïve**  **Mean ± SD**  **(n=43)** | **Male Trainer**  **(n=54)** | **FN vs FT** | **FN Vs MN** | **FT Vs MT** | **MN Vs MT** |
| Physiological | Age | 39.07±8.57 | 37.23±12.76 | 44.78±9.95 | 46.37±12.41 | 0.932 | 0.199 | 0.005 | 0.925 |
|  | Weight | 71.52±15.57 | 59.44±9.05 | 78.23±12.88 | 70.80±11.23 | **0.002** | 0.145 | **0.001** | **0.037** |
|  | BMI | 27.45±5.26 | 22.45±3.77 | 26.99±4.86 | 25.23±4.75 | **0.001** | 0.982 | 0.084 | 0.352 |
|  | POR | 86.93±10.51 | 93.56±14.83 | 86.94±13.82 | 91.10±15.42 | 0.315 | 1.00 | 0.892 | 0.765 |
|  | Systole | 115.78±14.62 | 119.51±14.03 | 123.87±10.17 | 126.60±11.35 | 0.211 | **0.002** | **0.004** | 0.187 |
|  | Diastole | 72.74±13.21 | 74.00±10.31 | 80.54±6.81 | 80.55±9.08 | 0.568 | **0.001** | **0.003** | 0.855 |
| Biochemical | TG | 142.74±84.87 | 99.90±43.75 | 174.54±82.40 | 122.56±63.06 | **0.030** | **0.039** | **0.033** | **<0.0001** |
|  | TC | 166.11±38.25 | 159.05±37.81 | 173.72±28.96 | 166.39±32.57 | 0.874 | 0.822 | 0.815 | 0.778 |
|  | HDL | 47.70±9.33 | 55.23±8.98 | 44.41±8.83 | 50.07±7.5 | **0.008** | 0.456 | 0.072 | **0.021** |
|  | LDL | 87.22±23.10 | 84.03±31.33 | 94.37±25.27 | 91.44±27.41 | 0.974 | 0.740 | 0.683 | 0.965 |
|  | VLDL | 28.56±17.02 | 20.05±8.84 | 34.94±16.51 | 24.93±12.56 | **0.035** | **0.038** | **0.021** | **0.001** |
|  | Ratio | 3.56±.73 | 2.87±.64 | 4.03±.81 | 3.37±.77 | **0.005** | 0.071 | **0.034** | **0.001** |
|  | Glucose | 99.63±23 | 112.80±26.74 | 103.54±22.98 | 108.94±25 | **0.017** | 0.109 | 0.546 | 0.487 |

|  | | | | **Age** | | **POR** | | | **Systolic** | | **Diastolic** | | **Weight** | | **BMI** | |
| --- | --- | --- | --- | --- | --- | --- | --- | --- | --- | --- | --- | --- | --- | --- | --- | --- |
| Age | | Pearson Correlation | | 1 | | -0.105 | | | 0.443 | | 0.280 | | 0.227 | | 0.153 | |
|  |  | Sig. (2-tailed) | |  | | 0.185 | | | <0.001 | | <0.001 | | 0.004 | | 0.054 | |
| POR | | Pearson Correlation | | -0.105 | | 1 | | | -0.055 | | 0.032 | | -0.170 | | -0.210 | |
|  |  | Sig. (2-tailed) | | 0.185 | |  | | | 0.496 | | 0.692 | | 0.031 | | 0.008 | |
| Systolic | | Pearson Correlation | | 0.443 | | -0.055 | | | 1 | | 0.607 | | 0.252 | | 0.203 | |
|  |  | Sig. (2-tailed) | | <0.001 | | 0.496 | | |  | | <0.001 | | 0.001 | | 0.011 | |
| Diastolic | | Pearson Correlation | | 0.280 | | 0.032 | | | 0.607 | | 1 | | 0.260 | | 0.171 | |
|  |  | Sig. (2-tailed) | | <0.001 | | 0.692 | | | <0.001 | |  | | 0.001 | | 0.033 | |
| Weight | | Pearson Correlation | | 0.227 | | -0.170 | | | 0.252 | | 0.260 | | 1 | | 0.833 | |
|  |  | Sig. (2-tailed) | | 0.004 | | 0.031 | | | 0.001 | | 0.001 | |  | | <0.001 | |
| BMI | | Pearson Correlation | | 0.153 | | -0.210 | | | 0.203 | | 0.171 | | 0.833 | | 1 | |
|  |  | Sig. (2-tailed) | | 0.054 | | 0.008 | | | 0.011 | | 0.033 | | <0.001 | |  | |
|  | | | | | | | | | | | | | | | | |
|  | | | **Age** | | **Glucose** | | **TG** | **TC** | | **HDL** | | **LDL** | | **VLDL** | | **Ratio** |
| Age | Pearson Correlation | | 1 | | 0.204 | | 0.226 | 0.189 | | 0.038 | | 0.096 | | 0.218 | | 0.155 |
|  | Sig. (2-tailed) | |  | | 0.013 | | 0.004 | 0.016 | | 0.631 | | 0.226 | | 0.006 | | 0.049 |
| Glucose | Pearson Correlation | | 0.204 | | 1 | | 0.147 | 0.000 | | 0.056 | | -0.091 | | 0.144 | | -0.034 |
|  | Sig. (2-tailed) | | 0.013 | |  | | 0.075 | 0.999 | | 0.500 | | 0.271 | | 0.083 | | 0.686 |
| TG | Pearson Correlation | | 0.226 | | 0.147 | | 1 | 0.421 | | -0.299 | | 0.050 | | 0.994 | | 0.606 |
|  | Sig. (2-tailed) | | 0.004 | | 0.075 | |  | <0.001 | | <0.001 | | 0.526 | | <0.001 | | <0.001 |
| TC | Pearson Correlation | | 0.189 | | 0.000 | | 0.421 | 1 | | 0.215 | | 0.872 | | 0.435 | | 0.597 |
|  | Sig. (2-tailed) | | 0.016 | | 0.999 | | <0.001 |  | | 0.006 | | <0.001 | | <0.001 | | <0.001 |
| HDL | Pearson Correlation | | 0.038 | | 0.056 | | -0.299 | 0.215 | | 1 | | 0.094 | | -0.301 | | -0.573 |
|  | Sig. (2-tailed) | | 0.631 | | 0.500 | | <0.001 | 0.006 | |  | | 0.234 | | <0.001 | | <0.001 |
| LDL | Pearson Correlation | | 0.096 | | -0.091 | | 0.050 | 0.872 | | 0.094 | | 1 | | 0.066 | | 0.576 |
|  | Sig. (2-tailed) | | 0.226 | | 0.271 | | 0.526 | <0.001 | | 0.234 | |  | | 0.407 | | <0.001 |
| VLDL | Pearson Correlation | | 0.218 | | 0.144 | | 0.994 | 0.435 | | -0.301 | | 0.066 | | 1 | | 0.619 |
|  | Sig. (2-tailed) | | 0.006 | | 0.083 | | <0.001 | <0.001 | | <0.001 | | 0.407 | |  | | 0.001 |
| Ratio | Pearson Correlation | | 0.155 | | -0.034 | | 0.606 | 0.597 | | -0.573 | | 0.576 | | 0.619 | | 1 |
|  | Sig. (2-tailed) | | 0.049 | | 0.686 | | <0.001 | <0.001 | | <0.001 | | <0.001 | | <0.001 | |  |

Table S4: Pearson’s bivariate analysis showing the correlation of age with physiological and biochemical parameters in naive and trainer participants.

|  | | | | **Yoga Training** | | **POR** | | **Systolic** | | **Diastolic** | | | **Weight** | | **BMI** | |
| --- | --- | --- | --- | --- | --- | --- | --- | --- | --- | --- | --- | --- | --- | --- | --- | --- |
| **Yoga Training** | | Pearson Corr | | 1 | | 0.075 | | 0.000 | | 0.075 | | | 0.092 | | 0.019 | |
|  |  | Sig. (2-tailed) | |  | | 0.510 | | 1.000 | | 0.521 | | | 0.421 | | 0.868 | |
| **POR** | | Pearson Corr | | 0.075 | | 1 | | -0.055 | | 0.032 | | | -0.170 | | -0.210 | |
|  |  | Sig. (2-tailed) | | 0.510 | |  | | 0.496 | | 0.692 | | | 0.031 | | 0.008 | |
| **Systolic** | | Pearson Corr | | 0.000 | | -0.055 | | 1 | | 0.607 | | | 0.252 | | 0.203 | |
|  |  | Sig. (2-tailed) | | 1.000 | | 0.496 | |  | | <0.001 | | | 0.001 | | 0.011 | |
| **Diastolic** | | Pearson Corr | | 0.075 | | 0.032 | | 0.607 | | 1 | | | 0.260 | | 0.171 | |
|  |  | Sig. (2-tailed) | | 0.521 | | 0.692 | | <0.001 | |  | | | 0.001 | | 0.033 | |
| **Weight** | | Pearson Corr | | 0.092 | | -0.170 | | 0.252 | | 0.260 | | | 1 | | 0.833 | |
|  |  | Sig. (2-tailed) | | 0.421 | | 0.031 | | 0.001 | | 0.001 | | |  | | <0.001 | |
| **BMI** | | Pearson Corr | | 0.019 | | -0.210 | | 0.203 | | 0.171 | | | 0.833 | | 1 | |
|  |  | Sig. (2-tailed) | | 0.868 | | 0.008 | | 0.011 | | 0.033 | | | <0.001 | |  | |
|  | | | | | | | | | | | | | | | | |
|  | | | **Yoga Training** | | **Glucose** | | **TG** | | **TC** | | **HDL** | **LDL** | | **VLDL** | | **Ratio** |
| **Experience** | Pearson Corr | | 1 | | -0.014 | | 0.094 | | 0.230 | | 0.012 | 0.237 | | 0.089 | | 0.204 |
|  | Sig. (2-tailed) | |  | | 0.911 | | 0.408 | | 0.041 | | 0.915 | 0.035 | | 0.436 | | 0.072 |
| **Glucose** | Pearson Corr | | -0.014 | | 1 | | 0.147 | | 0.000 | | 0.056 | -0.091 | | 0.144 | | -0.034 |
|  | Sig. (2-tailed) | | 0.911 | |  | | 0.075 | | 0.999 | | 0.500 | 0.271 | | 0.083 | | 0.686 |
| **TG** | Pearson Corr | | 0.094 | | 0.147 | | 1 | | 0.421 | | -0.299 | 0.050 | | 0.994 | | 0.606 |
|  | Sig. (2-tailed) | | 0.408 | | 0.075 | |  | | <0.001 | | <0.001 | 0.526 | | <0.001 | | <0.001 |
| **TC** | Pearson Corre | | 0.230 | | 0.000 | | 0.421 | | 1 | | 0.215 | 0.872 | | 0.435 | | 0.597 |
|  | Sig. (2-tailed) | | 0.041 | | 0.999 | | <0.001 | |  | | 0.006 | <0.001 | | <0.001 | | <0.001 |
| **HDL** | Pearson Corr | | 0.012 | | 0.056 | | -0.299 | | 0.215 | | 1 | 0.094 | | -0.301 | | -0.573 |
|  | Sig. (2-tailed) | | 0.915 | | 0.500 | | <0.001 | | 0.006 | |  | 0.234 | | <0.001 | | <0.001 |
| **LDL** | Pearson Corr | | 0.237 | | -0.091 | | 0.050 | | 0.872 | | 0.094 | 1 | | 0.066 | | 0.576 |
|  | Sig. (2-tailed) | | 0.035 | | 0.271 | | 0.526 | | <0.001 | | 0.234 |  | | 0.407 | | <0.001 |
| **VLDL** | Pearson Corr | | 0.089 | | 0.144 | | 0.994 | | 0.435 | | -0.301 | 0.066 | | 1 | | 0.619 |
|  | Sig. (2-tailed) | | 0.436 | | 0.083 | | <0.001 | | <0.001 | | <0.001 | 0.407 | |  | | <0.001 |
| **Ratio** | Pearson Corr | | 0.204 | | -0.034 | | 0.606 | | 0.597 | | -0.573 | 0.576 | | 0.619 | | 1 |
|  | Sig. (2-tailed) | | 0.072 | | 0.686 | | <0.001 | | <0.001 | | <0.001 | <0.001 | | <0.001 | |  |

Table S5: Tabular representation of correlation of yogic duration with various anthropometric and

biochemical parameters in naive and trainer participants by Pearson’s correlation analysis.


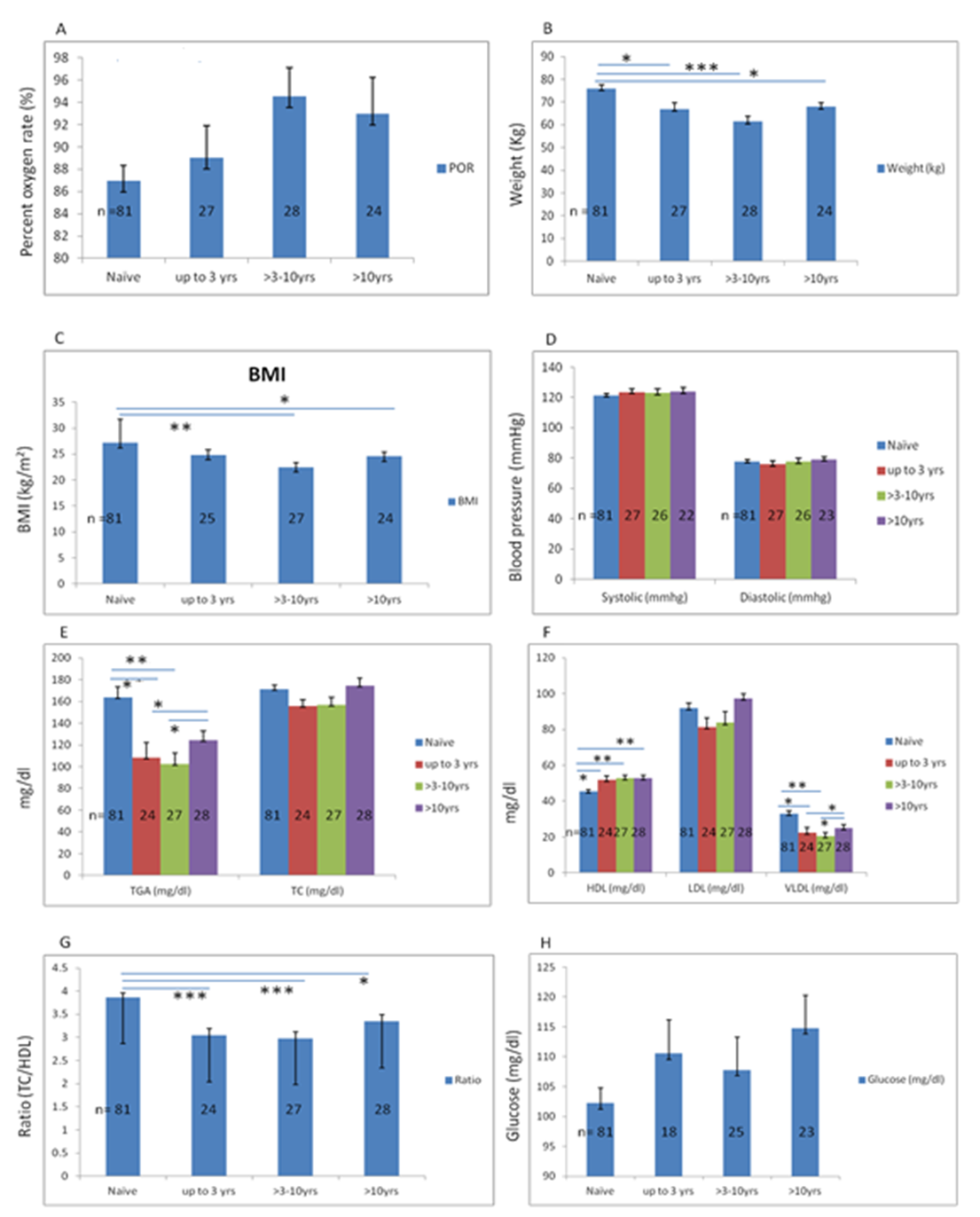
Figure S1


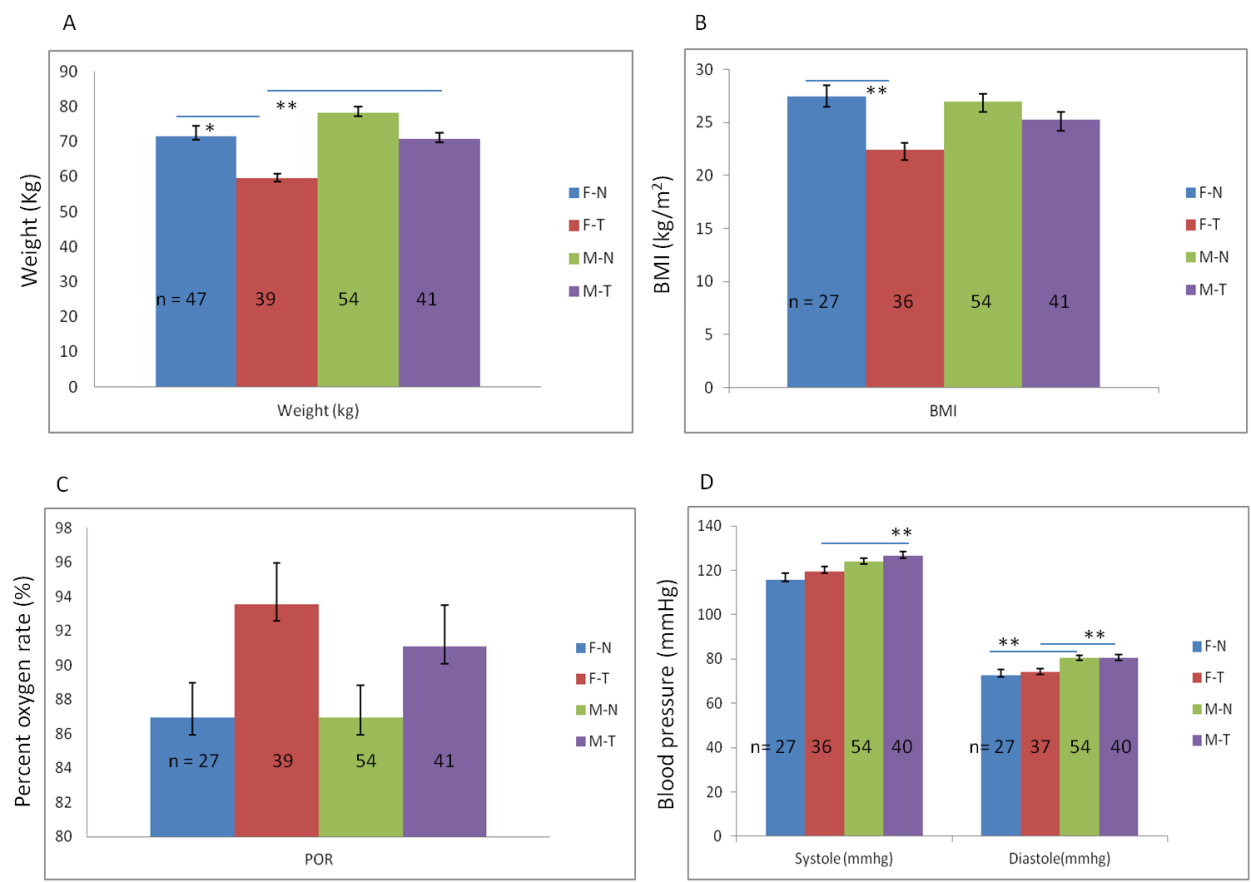


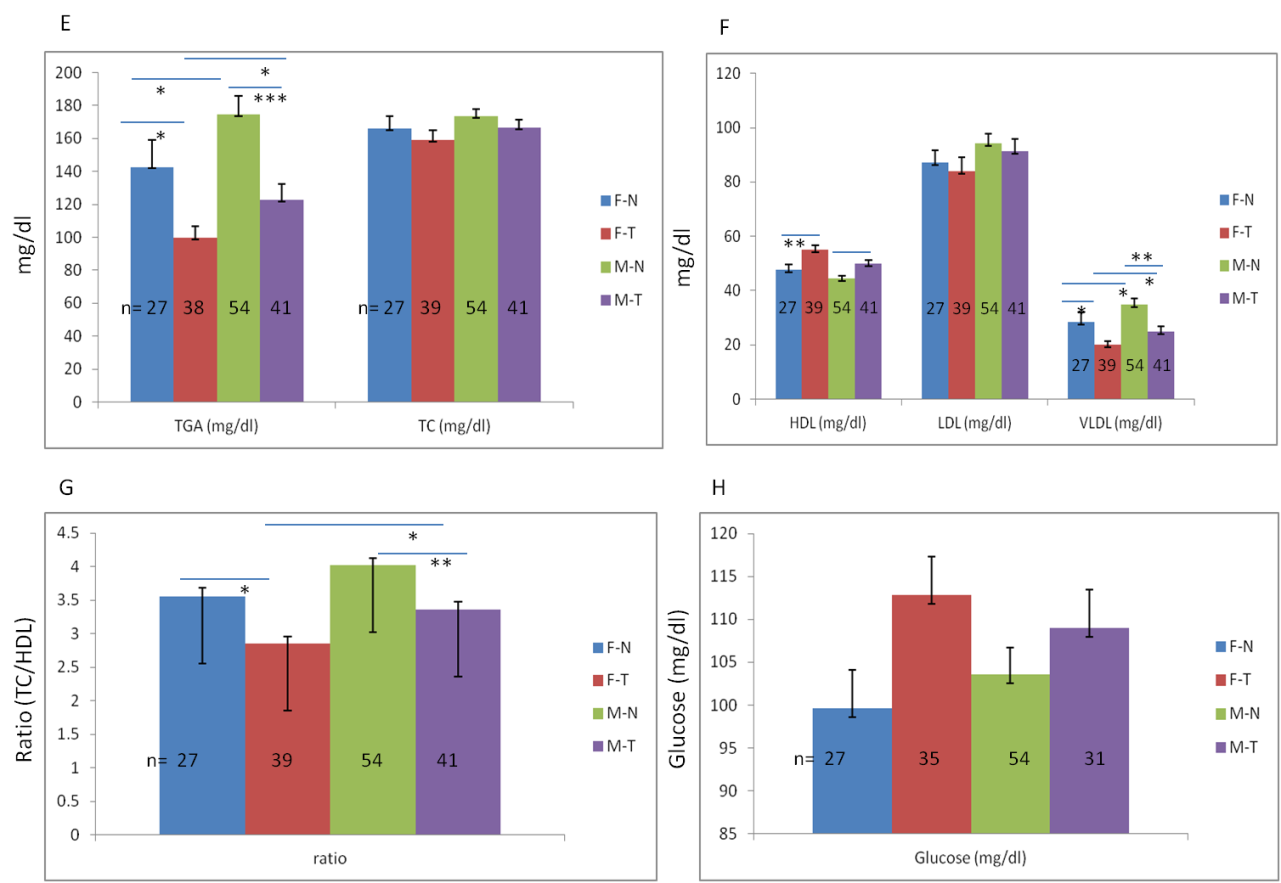


Figure S2

**Legends**

**Table S1**: Summary of yoga based on AYUSH Common Yoga Protocol 2016.

**Table S2**: Mean (with SD) and statistical significance in various physiological and biochemical parameters between different groups based on their age range.

**Table S3**: Tabular representation of gender wise comparison of physiological and biochemical parameters before and after yoga exposure in naïve and trainer participants.

Table S4: Pearson’s bivariate analysis showing the correlation of age with physiological and biochemical parameters in naive and trainer participants.

Table S5: Tabular representation of correlation of yogic duration with various anthropometric and biochemical parameters in naive and trainer participants by Pearson’s correlation analysis.

**Figure S1:** **Longitudinal and effective time of yoga practices to alter the physiological and biochemical parameters in the trainer group and comparison with naïve participants.** (A) Long term effect of yogic practice induces significant alteration in POR in >3-10 years group as compared to the yoga naïve group. (B & C) Weight and BMI analysis of trainer participants show a signifiant réduction in comparison to naïve participants with the duration (in years) of yoga practice but did not show any significant changes in blood pressure (D). (E) Components of lipid metabolism and glucose were also found to be altered with the yoga practice duration in the trainer group as compared to yoga participants. Significant changes in TG levels were observed in the trainer group, as well as in comparison to yoga participants. However, significant changes in TC levels within trainer group and also in comparison to naive were not observed. (F) HDL levels were found to be increased in all trainer groups in comparison to naïve participants and didn’t show any difference among the trainer group. Similarly, VLDL levels were found to be decreased in the trainer group in comparison to the naïve participants. Moreover, significant changes in VLDL levels have also been observed in >\10 years trainer group as compared to the remaining two (i.e. up to three years and 3-10years). That may be due to the age of the participants, although reduced levels of LDL have also been observed. (G) The ratio of TC and HDL was also significantly reduced in all trainer groups as compared to naïve groups. (H) Moreover, glucose levels (mg/dl) also increased in all trainer groups as compared to naïve groups, but the more significant difference was found between the naïve and >10years groups. However, it was noted in most of trainer groups that all physiological as well as biochemical parameters were increased in >10 years in comparison to the remaining two groups, i.e. up to 3 and 3-10 years groups. That may be due to the increase in age of >10 years group. * statistical significance ≤0.05; ** statistical significance 0.001 and *** statistical significance <0.0001. Bar is showing standard error mean (SEM).

**Figure S2: Gender-wise effects of yogic practices. Results revealed that females can adopt yogic practices more easily and also can attain homeostasis early by regulating both physiological and biochemical parameters (including hormonal and metabolic processes) in comparison to male.** (A & B) Weight and BMI both were significantly reduced in trainer females as compared to naïve females. Even female trainers were found to be reduced in weight and BMI as compared to trainer males. (D) Blood pressure (systolic and diastolic) was significantly altered in trainer males and females as compared to naïve males and females. (C) However, increased percent O_2_ saturation was found in female and male trainers, but was not statistically significant. (E) Biochemical analysis revealed that TG levels in serum were significantly altered in female and male trainers as compare to respective naïve participants. Moreover, changes in levels of TG were significantly more in female trainers as compared to male trainers. However, any significant alteration in total cholesterol levels (mg/dl) between any groups was not found. (F) Female trainers showed increased HDL levels as compared to naïve female participants. However, the base levels of HDL in naïve females were higher than naïve males. Additionally, the male trainers were found to have decreased levels of HDL as compared to female trainers. Similarly, VLDL levels were found to be decreased in female trainers as compared to naïve female participants. Results have also shown that the base levels of VLDL were significantly reduced in naïve female as compared to the naïve male group. Moreover, changes in VLDL levels in trainer females were found to be significantly less than the male trainer group. However, significant changes were not found in LDL levels between any groups. (G) Results obtained from ratio (TC to HDL) were also concomitant with the results as shown in VLDL. (H) Glucose levels in female trainers were significantly higher as compared to male trainer. *statistical significant ≤0.05; ** statistical significance 0.001 and *** statistical significance <0.0001. Bar is showing standard error mean (SEM).
